# Functional Rehabilitation in Non-Reconstructed Hemimandibulectomy Patients

**DOI:** 10.3390/medicina60121931

**Published:** 2024-11-23

**Authors:** Edoardo Brauner, Federico Laudoni, Emilia Rampelli, Andrea Bellizzi, Francesca De Angelis, Nicola Pranno, Andrea Battisti, Valentino Valentini, Stefano Di Carlo

**Affiliations:** 1 Department of oral and Maxillofacial Sciences, Sapienza University of Rome, Via Caserta 6, 00161 Rome, Italy; 2 Implanto-Prosthetic Unit, Policlinico Umberto I, Viale Regina Elena 287b, 00161 Rome, Italy; 3 Oncological and Reconstructive Maxillo—Facial Surgery Unit, Policlinico Umberto I, Viale del Policlinico 155, 00161 Rome, Italy

**Keywords:** hemimandibulectomy, maxillofacial surgery, dental implant, prosthetic rehabilitation, occlusal plane

## Abstract

*Background and Objectives:* Functional rehabilitation in patients with hemimandibulectomy remains a challenge no matter what method of reconstruction is chosen by physicians. In this paper, we aim to evaluate the feasibility of an acceptable occlusal restoration in patients who have undergone hemimanidublectomy without the reestablishment of mandibular continuity. *Materials and Methods:* Data were collected retrospectively on 10 patients with varying degrees of mandible resection. The greatest challenge in the restoration of an acceptable occlusion form is the natural latero-deviation that occurs in patients whose mandibular continuity was not restored. This causes an unbalanced and uncoordinated occlusal plane. Based on our research, this deviation is highly dependent on three main factors: the presence of teeth (which allow for a proprioceptive stimulus that counteracts the deviation), the extension of the defect and the presence or lack of the coronoid process. *Results:* Based on the presence of dental elements or lack thereof, patients were either rehabilitated with implant-supported dentures or removable partial dentures. Although the gold standard of care for these patients remains the restoration of mandible continuity through use of bone grafts, an acceptable rehabilitation of occlusion and therefore function may be acquired in non-grafted patients. *Conclusions:* Both physicians and patients must manage their expectations carefully and be eager to find a compromise to provide the best possible solution given the challenges of the premises.

## 1. Introduction

The Italian association of medical oncology (AIOM) estimates that there were over 9900 new cases of cancer of the oral cavity in 2020 in Italy, of which approximately 4100 will succumb to the disease. In 2020, oral cancer was estimated to rank 16th in incidence and mortality worldwide [1,2].

In addition to the threat to life, the insurgence of oral cancer can lead to several deficiencies of various levels of gravity regarding oral functions—talking, swallowing, and chewing—as well as an alteration of one’s perceived physical appearance and chronic pain. These collectively contribute to a sharp decline in quality of life [3].

Surgery remains the standard care practice for oral cancer; while early-stage tumors can be treated with a relatively noninvasive surgical resection that rarely requires major reconstructive effort, advanced-stage cancers command major ablative surgery followed by reconstructive surgery and adjuvant therapy [4].

Advanced tumors that involve the mandible require resection of the mandible (marginal or segmental mandibulectomy). Segmental mandibular resection is perhaps one of the most pivotal choices to be made in the management of oral cancer: based on the extent and orientation of the resected portion of the mandible, reconstruction can become more complex and more essential.

Mandible reconstruction represents a key step in the rehabilitation of patients that undergo mandible ablative surgery. The defects can be marginal (resection of a single cortex of the mandible) or segmental (removal of an entire segment, including both cortices and medullary space). Segmental mandibulectomy results in a loss of continuity that can be restored with osteocutaneous free flaps, which can be obtained from a variety of donor sites (fibula, iliac crest, radius, etc.) [5].

The functional and aesthetic outcomes are more successful when osteocutaneous flaps are implemented [6]. The fibula free flap is one of the most widely implemented flaps thanks to its long pedicle length, relative ease of modification of form (which improves its versatility) and suitability as a recipient site for implants [5].

The fibular flap represents the standard therapy choice for anterior segmental defects, as it supports chin projection and facial width while also maintaining a relatively low complication rate [7].

However, the fibular flap does display some limitations. According to Mosahebi et al., the stiffness of the skin does not allow adequate molding, which is of paramount importance in the reconstruction of large surface area defects, such as the posterior lateral area of the oropharynx. In addition to this, following ablative surgery, soft-tissue-related loss of volume is often present, and the fibular osteocutaneous flap provides insufficient bulk [8]. Furthermore, the masticatory muscles are detached from the mandible in most posterior resections, further lessening the debatable benefits of vascularized bone flap reconstruction.

Extensive composite defects of the oromandibular area that involve the skin, mandible, oral mucosa, and soft tissues require complex reconstructive procedures: flaps with adequate soft tissue are needed to fill the extensive space left by huge oromandibular defects. However, these kinds of flaps also tend to sink and droop with time due to gravity, resulting in poor functional and aesthetic results, especially when support from the mylohyoid muscle is lacking [9].

The aim of this study is to ascertain that, despite major lateral deviation, the restoration of a correct occlusion form and vertical dimension is of relevant importance and can be achieved in the rehabilitation of oral functions in patients with only soft tissue reconstruction or primary wound closure.

## 2. Materials and Methods

This study is a single-institution, retrospective review. Patients who underwent reconstruction of composite oromandibular defects following mandibulectomy were identified. Patients who underwent soft tissue restoration of a bony mandibular defect with or without adjacent soft tissue resection were identified and considered eligible for assessment, along with patients whose resection sites were sealed by primary intention healing. Those patients who underwent osseous reconstruction with vascularized bone flaps were excluded from this study. Further inclusion criteria are complete clinical and radiological documentation and a complete prosthetic rehabilitation at the Implantoprosthesis Unit of the Head and Neck department in Sapienza University of Rome.

At present, there exists no universally accepted classification system of mandibular defects. Amongst the most cited in the literature is the HCL classification [10], which has seen widespread use thanks to its versatility and intuitiveness. In 2016, Brown at al. introduced a new classification based on the 4 corners of the mandible (2 vertical corners that make the angles of the mandible, and 2 horizontal corners which find their center at the canine teeth). These corners represent the fulcrum of change in the shape of the mandible and the consequent increasing need to shape a graft with osteotomies [10,11].

The classification used for this study is the 2016 Brauner et al. classification, which aims to correlate the anatomical defect with the most appropriate prosthetic rehabilitation [12].

A total of 20 patients with hemimandibulectomy were identified from the department database. Of these patients, 3 were excluded from the present study due to osseous reconstruction, 1 was excluded due to a partial mandibulectomy (non-segmental), 4 were excluded due to insufficient documentation and 2 did not complete the rehabilitation treatment. In total, 10 patients met the inclusion criteria and were therefore included in the present study (Table 1).

## 3. Results

### 3.1. Sample Characteristics

All the patients underwent surgery due to squamous cell carcinoma. Most of the patients were male, with a mean age of 69 years old. Three patients had a fixation plate inserted during the surgery, to stabilize the remaining segments of the lower jaw. Six patients underwent radiation therapy post operation, while one underwent chemotherapy, and one patient underwent adjuvant therapy of both kinds. Four out of eleven of the studied patients were edentulous at the time of treatment.

### 3.2. Surgical Treatment Details

All 10 patients were treated for the excision of tumoral tissue at the Department of Maxillofacial Surgery of the Umberto I hospital. Most of the surgeries (9 out of 11 surgeries) included selective or radical neck dissection, depending on the tumoral stage and risk of lymph node metastases. One patient’s surgical treatment included tonsillectomy, while nine patients’ excision surgery included tracheotomy, trans- or sub-isthmic, based on an assessment of risk factors of postoperative airway obstruction.

Based on extension of affection of soft tissue, two patients underwent resection of the buccalpelvis tissue, and one patient was subjected to excision of one tonsil pillar. One patient was subjected to glossopelvectomy in addition to hemimandibulectomy.

Following surgery, most patients underwent irradiation therapy. This was mostly because, following a global trend, most patients affected by squamous cell carcinoma (SCC) have a negative prognosis due to a diagnostic delay. This in turn causes physicians to face, for the most part, advanced cases of SCC which require aggressive adjuvant therapy such as irradiation.

### 3.3. Dental Rehabilitation Details

All patients but one were initially rehabilitated with a partially or totally removable prosthesis (based on the dental status of each individual patient) to recover both function (phonation, deglutition, mastication) and aesthetic. The first prosthesis also serves as a guide plane to correctly orient the remaining teeth during function, minimizing the natural latero-deviation that occurs in these patients. Four patients decided to continue treatment with a final implant-retained prosthesis.

Two explanatory cases of implant rehabilitation are shown in cases 1 and 2, while three cases of removable prosthesis are shown in cases 3–5.

**Figure 1:**
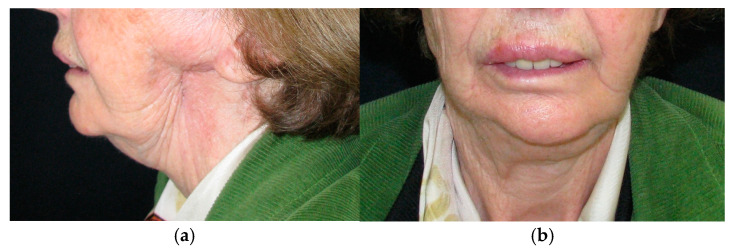
Patient with hemimandibulectomy L2 in lateral (**a**) and front (**b**) extraoral view.

**Figure 2:**
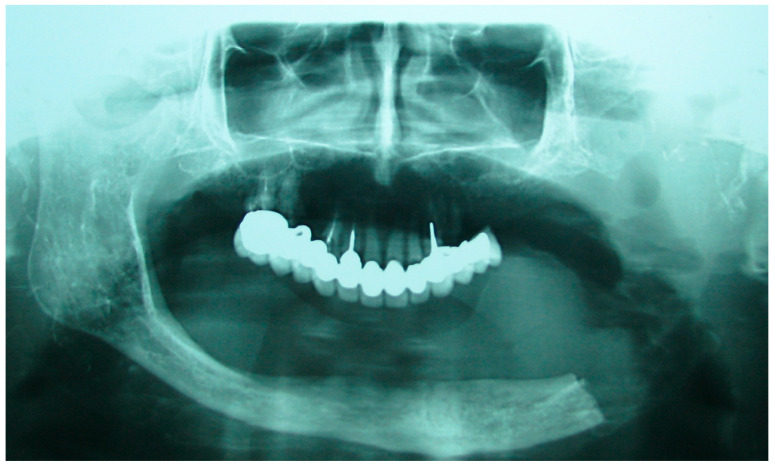
Patient with hemimandibulectomy L2 in orthopanoramic X-ray.

**Figure 3:**
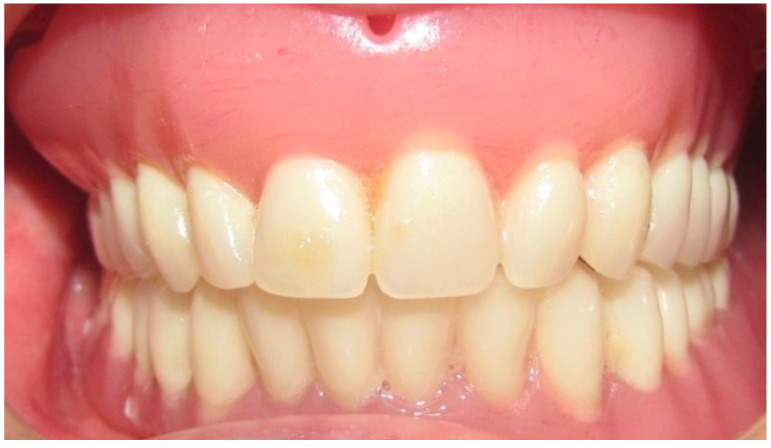
Transitional complete removable denture.

**Figure 4:**
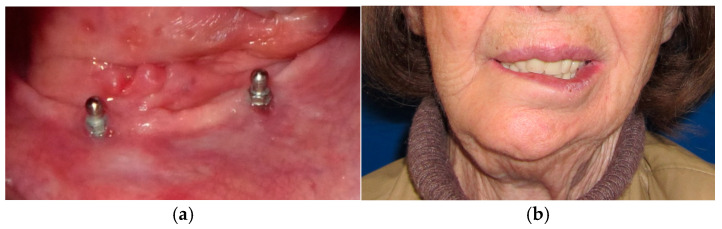
Intraoral view of implant placement in 3.2 and 4.4 area (**a**) and final prosthetic rehabilitation in extraoral view (**b**).

The patient in Case 1 had undergone surgery to remove a tumor that intersected the angle of the mandible. (L2) (Figure 1 and Figure 2). The upper elements were extracted soon after due to periodontal and endodontic issues. After being initially rehabilitated with a complete removable prosthesis (to regain function and aesthetic), two implants were subsequently placed in the 3.2 and 4.4 areas, and the final prosthesis was built in resin with a ball retention mechanism (Figure 3 and Figure 4).

The mandibular resection of this patient was not massive; hence, the rehabilitation project was in essence very similar to that of a normal edentulous patient, with the only exception of a rather extensive flange on the resected area to help stabilize the prosthesis during function.

**Figure 5:**
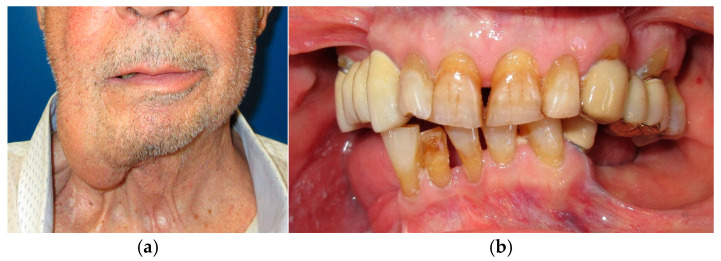
Patient with hemimandibulectomy L2, L3 in extra- (**a**) and intraoral (**b**) view.

**Figure 6:**
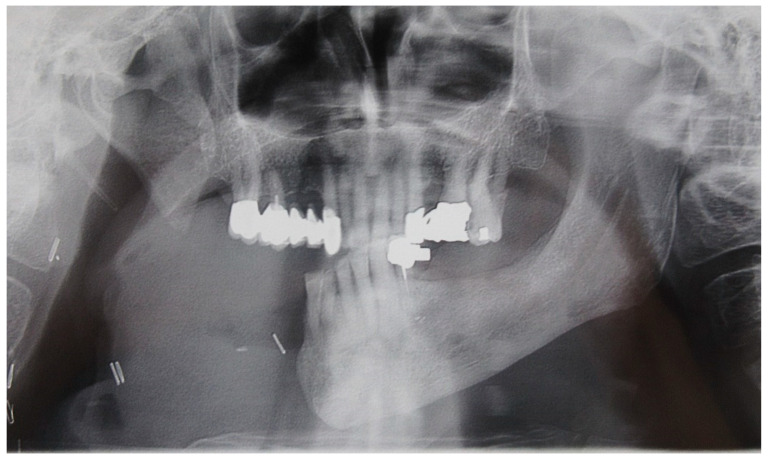
Patient with hemimandibulectomy L2, L3 in orthopanoramic X-ray.

**Figure 7:**
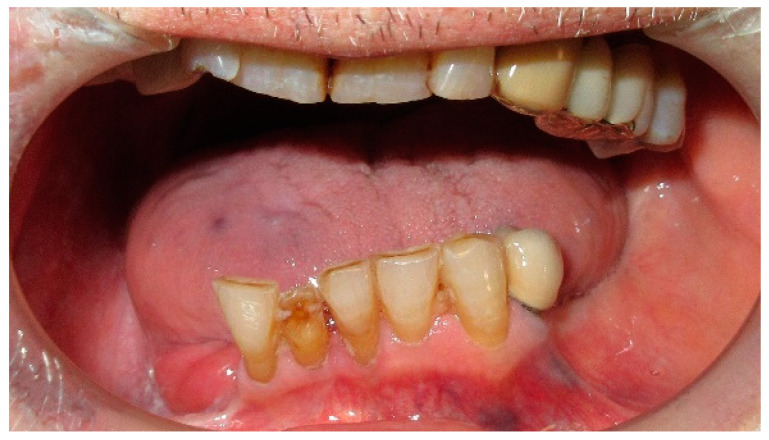
Intraoral view of the mouth opening.

The patient of case 2 presented an L2-3 resection, with preservation of both the condyle and the coronoid process (Figure 5 and Figure 6). Although posteriorly edentulous, the patient had maintained six lower elements (31, 32, 33, 34 (which hosts a prosthetic crown), 41, and 42). The trajectory between open and closed positions is roughly maintained, and the surviving mandible does not tend to swing laterally toward the surgical side during opening (Figure 7).

**Figure 8:**
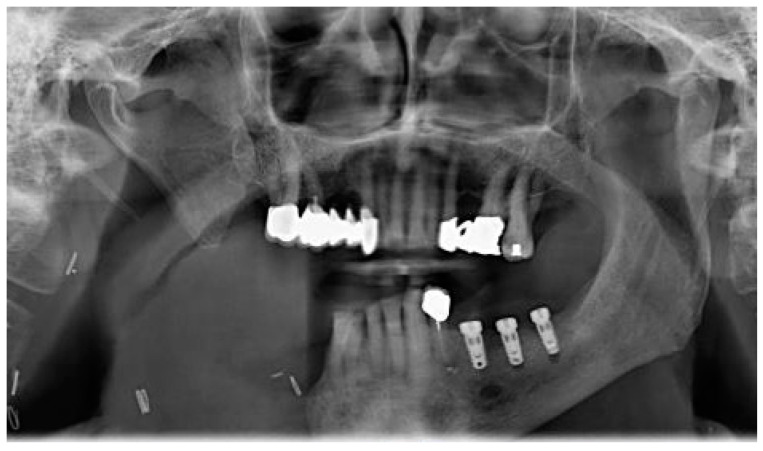
Rx view of three implants positioned in areas 3.4, 3.6, and 3.7.

**Figure 9:**
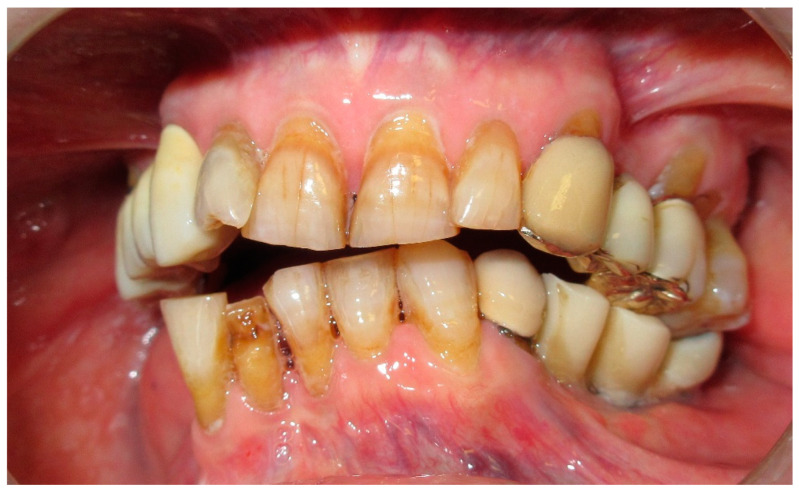
Intraoral view of implant-supported crowns in quadrant III.

He required no intermediate removable prosthesis. Although the mandibular resection resulted in a rather extensive bony defect, the placement of three implants in the III quadrant allowed for an intermediate rehabilitation with three prosthetic crowns (Figure 8 and Figure 9). The pictures regarding the result are not yet available, and the rehabilitation project is intended to develop as follows: the prosthetic crowns serve as an occlusal plane guide, helping the patient to find a more centric mandible position.

However, due to the rotational effect of these pivotal points, and the fact that the surviving lower teeth are too lingual to help stabilize the occlusal plane, we plan to extract the remaining lower teeth and potentially utilize GBR techniques to allow the customized placement of two additional implants in the symphysis area, which will in turn support prosthetic crowns, allowing further occlusal points.

The final prosthesis design will consist of a titanium primary structure to solidarize the five implants and a composite coated secondary structure which will include an extensive prosthetic flange on the area that was interested by surgery. The flange will include three to four dental elements, which will not serve a directly functional purpose (patients will be unable to chew on said side) but will help guide an acceptable interocclusal contact.

**Figure 10:**
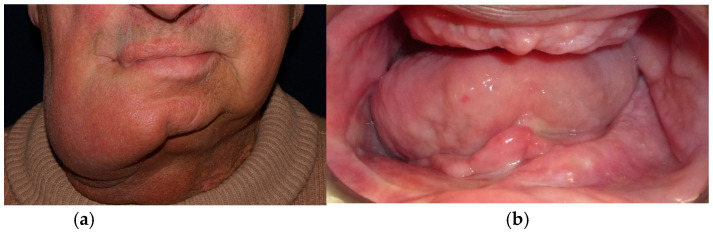
Patient with hemimandibulectomy L1,2,3 in extra- (**a**) and intra oral (**b**) view.

**Figure 11:**
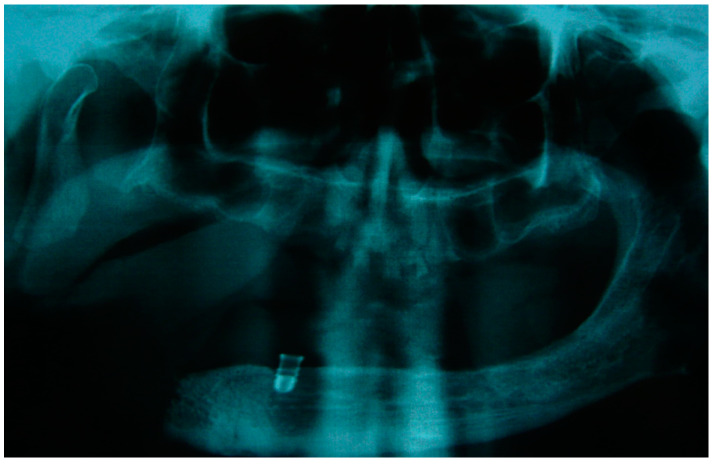
Patient with hemimandibulectomy L1,2,3 in orthopanoramic X-ray.

**Figure 12:**
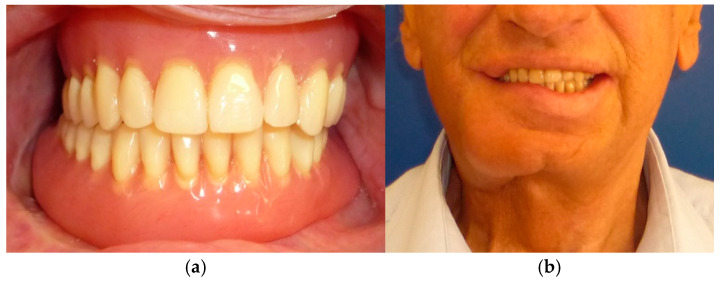
Final prosthetic rehabilitation in intra- (**a**,**b**) extraoral view.

The patient in case n°3 was rehabilitated with a complete removable prosthesis (Figure 10, Figure 11 and Figure 12). The patients’ implant (Figure 10), which was inserted in a different structure before the resection surgery took place, was removed following substantial evidence of periimplantitis that emerged following a TC cone beam analysis (not pictured). The patient underwent irradiation therapy post op and was supposed to be a candidate for implant rehabilitation; however, he chose to not continue with the treatment plan. The removable prosthesis, although not ideal due to the lack of a fixed structure to anchor the prosthesis on the surviving bony segment, did aid in guiding the mandible in a more centric and functional position.

**Figure 13:**
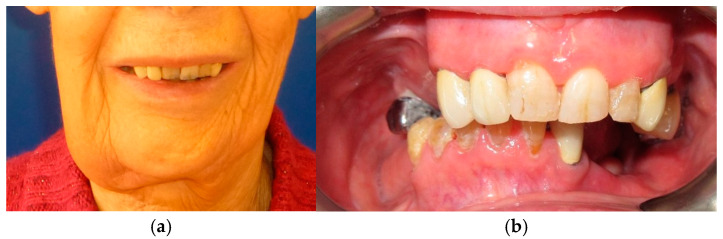
Patient with hemimandibulectomy L1,2,3 in extra- (**a**,**b**) intraoral view.

**Figure 14:**
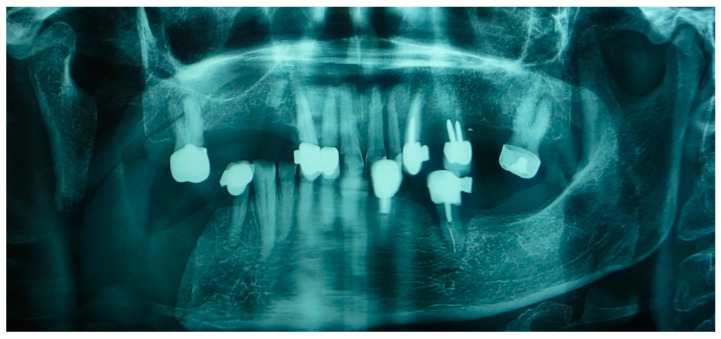
Patient with hemimandibulectomy L1,2,3 in orthopanoramic X-ray.

The patient in case n°4 underwent hemimandibulectomy resulting in an extensive bone defect (L1-2-3) that included the conoid process, the angle of the mandible and a portion of the body of the mandible (Figure 13 and Figure 14). Due to the defect, both functional movements of open and closure appear massively deviated: despite the presence of six dental elements (31, 32, and 33 treated endodontically and with a prosthetic crown and 41, 42, and 43), the lack of the coronoid process due to surgical resection does not allow for a more centered position of the mandible, as the surviving segment tends to deviate and pull massively towards the surgical side.

**Figure 15:**
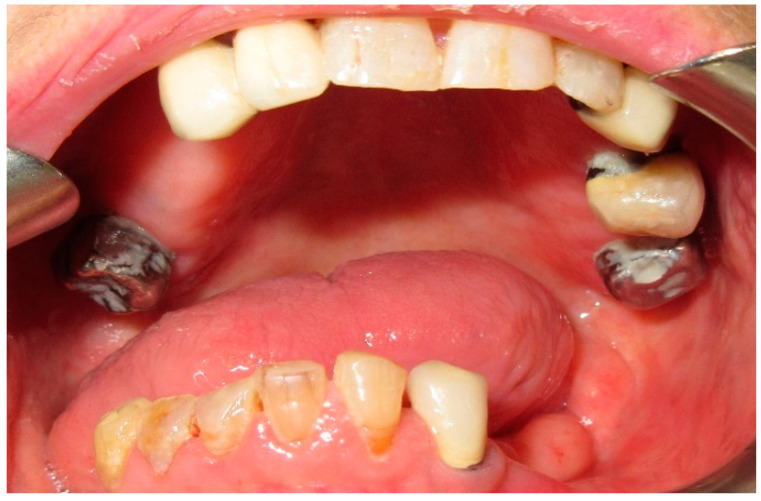
Intraoral view of the mouth opening.

The trajectory of the opening movement presents a further deviation. The deep bite probably represents a worsening factor for the deviation, due to the lack of proprioception (Figure 15).

**Figure 16:**
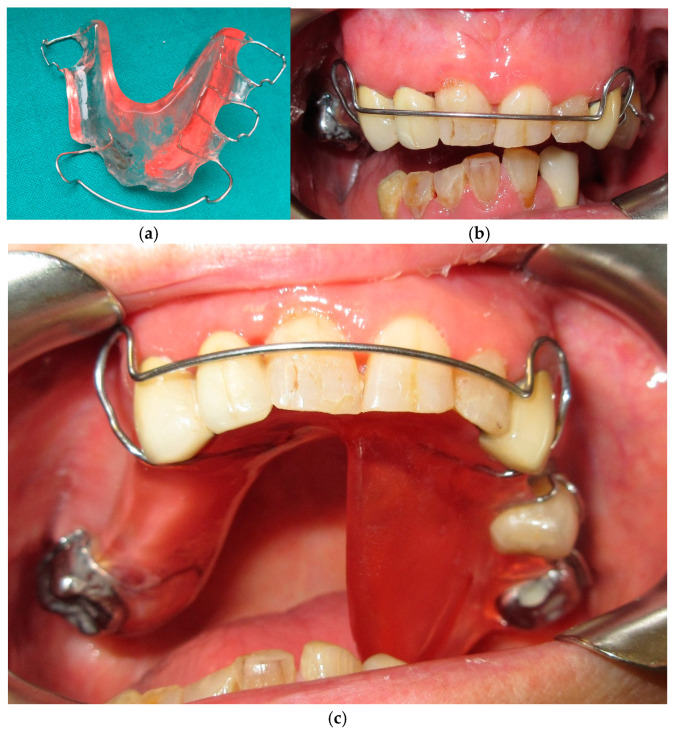
Intermediate occlusal plane guide in extraoral (**a**), front (**b**) and palatal (**c**) intraoral view.

**Figure 17:**
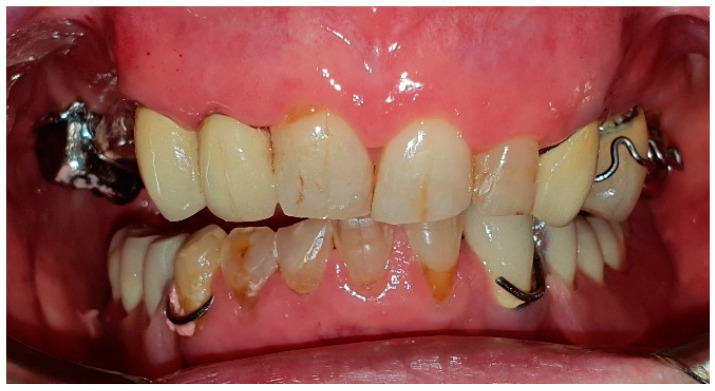
Occlusal plane guide and inferior removable prosthesis in intraoral view.

**Figure 18:**
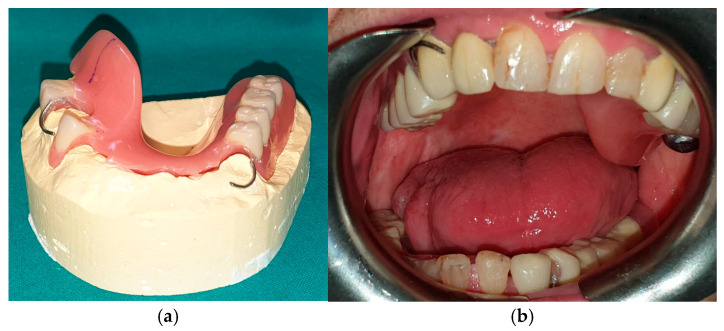
Definitive upper prosthesis with occlusal plane guide in extra- (**a**,**b**) intraoral view.

**Figure 19:**
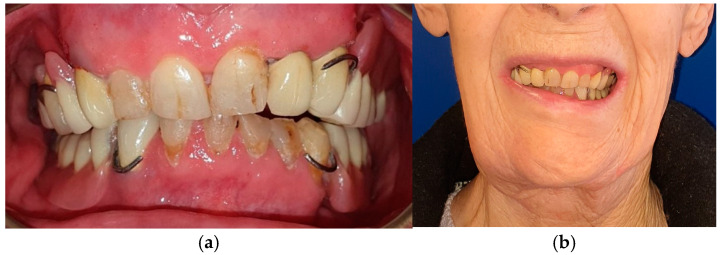
Final prosthetic rehabilitation in intra- (**a**,**b**) extraoral view.

The treatment plan included a transitional occlusal plane guide, an acrylic plate with labial bow for retention on upper teeth with a posterior acrylic process that extends inferiorly (Figure 16 and Figure 17). The contact between the occlusal surface of the remaining mandibular teeth and the acrylic process guides the remaining mandibular segment in a more centric position. This device can be considered a functional device, as it is a tool that works by stimulating the mandible to position itself in a more correct location by blocking the natural deviation thanks to the acrylic process that works as a brake against unwanted mandibular movement. The transitional occlusal plane was then developed in resin with composite teeth and an inferior removable prosthesis equipped with hooks (Figure 18 and Figure 19).

**Figure 20:**
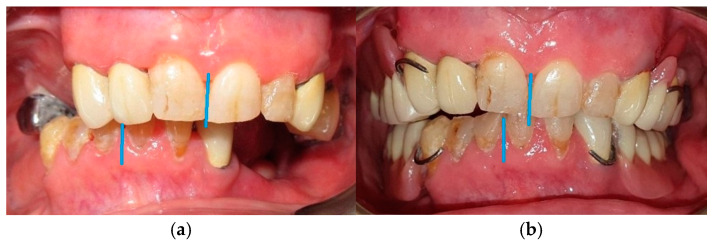
Intraoral view of before (**a**) and after (**b**) prosthetic rehabilitation. The blue line indicates the midline shift after prosthetic rehabilitation.

The comparison between the initial mandibular deviation and the result can be better explored in the following images (Figure 20).

**Figure 21:**
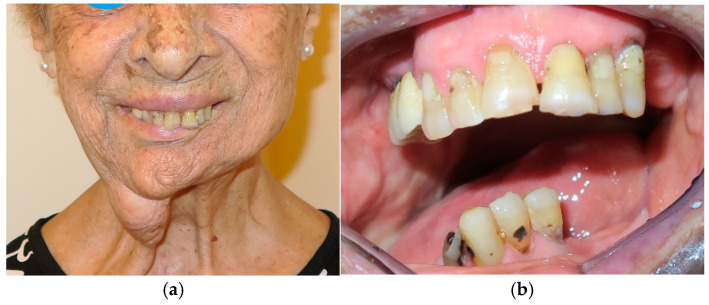
Patient with hemimandibulectomy L,2,3 in extra- (**a**,**b**) intraoral view.

**Figure 22:**
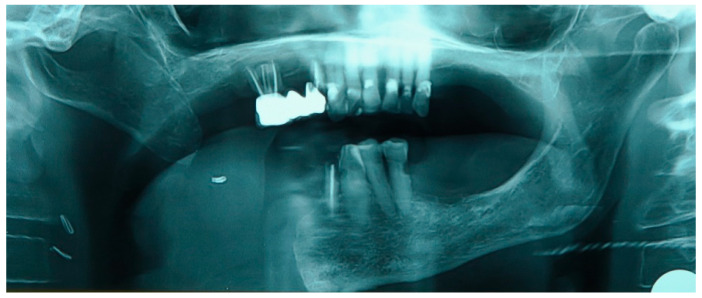
Patient with L2,3 hemimandibulectomy in orthopanoramic X-ray.

The patient of case 5 displayed a moderate latero-deviation following an L2-3 hemimandibulectomy with partial resection of the coronoid process (Figure 21 and Figure 22). Photos of interocclusal contact between the upper and lower teeth were not feasible due to the patient being unable to successfully close her mouth with the dental gag inserted.

The patient’s surviving teeth (32, 33, 34, 35) are not sufficient to stabilize and counteract the deviation towards the affected side, which not only develops on the transverse plane (left to right) but also on the frontal plane (back to front).

**Figure 23:**
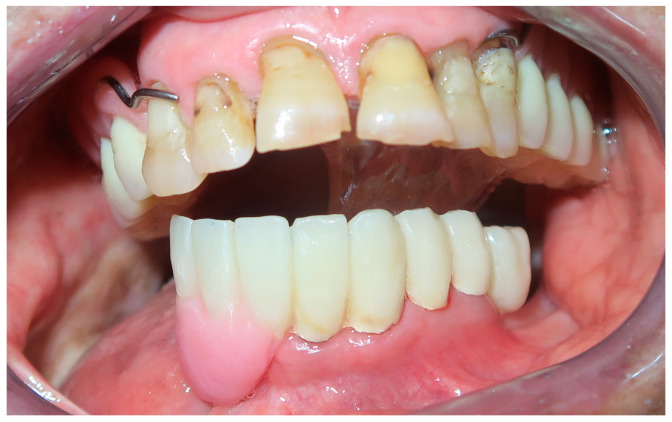
Transitional rehabilitation with superior removable prosthesis and fixed inferior prosthesis. The upper denture presents an acrylic process.

The presence of the lateral lower group of teeth (32, 33, 34, 35) was initially of pivotal importance, as it allowed for a temporary fixed prosthesis with the addition of four prosthetic elements (36, 31, 41, 42). This was coupled with a removable superior denture equipped with hooks and an acrylic process that projected downwards (Figure 23). In addition to improving function, the fixed prosthesis had the important goal of allowing more interdental contacts, which translated to an improved proprioceptive function. The latter, in addition to the acrylic process of the superior denture, helped the patient find a more adequate mandibular position. This solution was meant to be temporary, as the inferior posterior group of teeth still had to be rehabilitated for the patient to gain complete mastication function.

**Figure 24:**
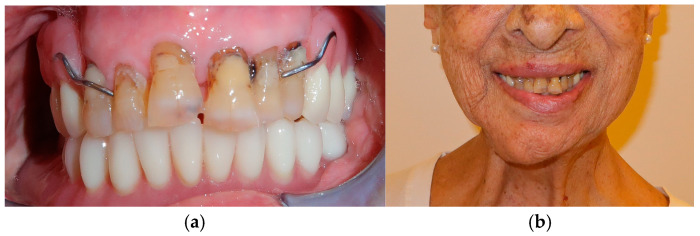
Final prosthetic rehabilitation in intra- (**a**,**b**) extraoral view.

The inferior teeth were subsequently treated endodontically and filed down. The choice of not extracting the surviving elements was made, keeping in mind the inevitable bone resorption that follows. The upper prosthesis was modified by removing the acrylic component, while in the lower prosthesis, the posterior elements were added to the frontal elements. This served the same purpose as the eliminated superior process, which was less functional and not meant as a long-time solution (Figure 24).

### 3.4. Follow-Up

The patients that were included in this study underwent surgery between 2007 and 2020. In 2023, they were contacted by phone for a series of follow-up questions that aimed to assess the long-time success or failure of the prosthetic rehabilitation they had received. Due to the frailty and advanced age of these patients, the questions were kept short and rather simple, and patients were invited to answer as truthfully as possible. The questions can be divided into three sections: adequacy of the prosthetic artifact (Is the patient able to wear the denture without pain or discomfort? Does it allow, if necessary, for an easy cleanse?), functionality (Does the artifact allow for an acceptable function regarding eating and speaking? Does it cause uneasiness or embarrassment to the patient in social situations?) and aesthetics (Is the patient satisfied with the aesthetic outcome?).

Of the 10 patients that took part in the study, 4 had passed away since the completion of rehabilitation and were hence not included in the follow-up. Of the interviewed patients, two were still in the process of rehabilitation (one patient was still at the complete removable denture phase of rehabilitation, waiting to undergo surgery to insert implants, while the other had inserted three implants and was waiting surgery for the insertion of an additional two implants). These two patients reported very different levels of satisfaction: the patient that had received the removable denture declared dissatisfaction with the functionality of the denture, as both functions of talking and eating were unsatisfactory. Furthermore, the patient reported embarrassment in social situations, and although the denture was not painful to wear, it did provide discomfort. The patient that was equipped with three implants that supported three crowns reported satisfaction in function, lack of embarrassment/discomfort in social situations, and lack of physical pain or discomfort. Both patients were satisfied with the aesthetic outcome, and they both found the artifact easy to cleanse. One patient, who had received a removable prosthetic artifact, had subsequently undergone secondary surgery to insert a reconstruction plate. The plate failed, as it suffered exposure after barely a year since the insertion. Since then, the patient stopped using the artifact that was provided by our department, on the advice of her surgeon, while waiting for her follow-up visit in the upcoming months. The patient reported dissatisfaction with the prosthetic artifact prior to and following the insertion of the plate in all but one section of the interview (aesthetic).

On average, patients that had been rehabilitated with fixed prosthesis (be it individual crowns on implants, or completely removable overdentures on implants) reported higher levels of satisfaction that were most evident in the functionality section compared to patients that were rehabilitated with removable artifacts, be it complete removable dentures or partial dentures equipped with hooks. This result is self-evident, as these patients have such unique oral characteristics that rarely allow for a suitable substrate for an acceptable removable denture.

## 4. Discussion

The surgical technique and modality are the result of the assessment of tumor factors (location, extent), patient factors (medical comorbidities, status of dentition and available bone and soft tissue, age, lifestyle), and physician-related factors (available expertise) [4,13].

In patients in whom it was necessary to include, during the surgical excision, a tracheotomy, trans- or sub-isthmic, it was evaluated based on the risk factors of postoperative airway obstruction such as the site of the primary tumor, the crossing of the median tumor size, preoperative radiotherapy, mandibulectomy, flap reconstruction, and neck dissection [14].

Radiation therapy better helps the prognosis of these patients while simultaneously being detrimental to the tissues that are directly involved. The short-term and immediately visible damage mostly concerns soft tissues and includes xerostomia and mucositis. This is particularly harmful in patients with soft tissue flaps. Other long-term impairment affects both soft tissue and hard tissue and includes hypo vascularization, cellular hypoxia, hypocellularity and fibrosis. These all contribute to causing a significant deficiency of the cellular reparative capacity processes, which in turn is associated with a lower resistance of the affected tissue to both trauma and infection [15,16].

The most obvious flaw of soft tissue free flap reconstruction is the inability to insert osseointegrated dental implants in the posterior mandibular region [13]. Additionally, in patients who have undergone mandibular extirpation, the remaining mandibular segment will retrude and deviate towards the surgical side, partly due to the absence of masticatory muscles on the affected side. This results in uncoordinated and therefore less efficient mandibular mobility, along with facial asymmetry. Function and aesthetics tend to worsen in edentulous patients due to impaired proprioception. Besides mandibular deviation, contact of the teeth on the non-surgical side increases the distance between any teeth on the surgical side and the opposing maxillary teeth. The remaining mandible tends to rotate in the frontal plane, with the occlusal contacts on the unresected side acting as a fulcrum [17,18].

Another significant flaw of the soft tissue flap, particularly with the pectoralis major flap, is its tendency to cause tension due to the bulkiness of the pedicle. This tension exacerbates mandibular deviation, as the flap’s tension pulls the surviving mandibular segment towards the surgical site.

Furthermore, our experience has shown that the coronoid process and the digastric muscle play a crucial role in mandibular deviation. Specifically, resection of the former generally results in lateral deviation (case 4), while involvement of the muscle leads to mandibular retrusion (case 5).

The use of bone grafts, especially the free fibula graft, offers significant advantages in implant positioning and optimal prosthetic rehabilitation when native bone is insufficient [19,20]. Several authors have advocated for immediate mandibular reconstruction after resection using vascularized free flaps, to prevent complications in implant placement following radiotherapy, as well as to enhance both facial symmetry and masticatory function [21,22].

According to Hanasono et al. (2010), the choice between vascularized bony free flap vs. soft tissue free flap must take into consideration the following factors: the extent and complexity of the defect, comorbidities, age, and adverse harvest environments. Soft tissue free flaps are the most fitting therapeutic choice used in advanced-age patients with unsuitable lower extremity conditions (e.g., severe leg edema) and significant medical comorbidities who display limited yet complicated mandibular defects. Bony flaps are to be chosen in patients whose defects extend anteriorly beyond the midline, given the patient donor site suitability, as these defects are mostly associated with loss of oral competence and decreased lower facial height. Ideally, vascularized bone flaps should be chosen for young and relatively healthy patients (no comorbidities) [13].

When implants are not feasible, a removable acrylic prosthesis with a guide flange offers a cost-effective alternative that also serves an active therapeutic function for the patient. Periodic adjustments to the acrylic guide flange gradually reduce its thickness, allowing the patient to eventually maintain proper occlusal alignment without the need for prosthetic support [23,24,25,26,27].

After total resection of the mobile tongue or more extensive resection, the tongue and oral floor must be reconstructed with sufficient height and roundness to make the oropharyngeal space narrow enough to restore glossopalatal closing function and regenerate swallowing pressure. Those patients need a mylohyoid muscle-like structure and neural anastomosis, with the goal of preventing fatty degeneration and muscle atrophy to maintain the bulge and prevent sinking of the reconstructed oral floor and tongue. Otherwise, the reconstructed oral floor and tongue cannot move and do not have sensation, and depression of the oral floor causes saliva and food residues to be trapped awkwardly, resulting in significant functional difficulties for patients [9].

The main factors that were considered in choosing the best rehabilitation strategy were the presence of teeth on the lower jaw and extension of the mandibular defect. It is important to note that, due to numerous variables that range from patient factors to surgical factors, there is no “one size fit all” rehabilitation pathway. Some patients, due to a massive deviation of the midline in open and closure positions, began their rehabilitation with an occlusal reeducation plate, which served the important purpose of guiding the mandible in a more functional and more centered position. Others, however, were able to maintain good muscular coordination and hence needed no orthopedic device to help them center the lower jaw.

The consequences of head and neck oncologic therapy have a negative influence on quality of life and affect all oral functions including chewing and phonation and, above all, the aesthetic of the face. Psychologically, a correct, valid rehabilitation allows the patient to become self-confident again; therefore, both social and work life improve. Optimal rehabilitation has a positive and powerful effect on the patient’s psyche, and it allows the start of a non-dysfunctional life [28,29].

Today, prosthetically guided rehabilitation represents the main rehabilitation protocol in prosthodontics, especially in those oncological patients with relevant loss of tissues and modified anatomy. Because of structures’ modifications, a simple mobile prosthesis can show less efficiency. Fixed prosthesis is the best option because it guarantees stability, reduces mucosal inflammation, and properly restores functional, mechanical, and aesthetic properties [29,30]. However, this is not always possible because it is necessary to consider several aspects such as bone availability, general health conditions, compliance, and patient expectations.

In evaluating the role of the coronoid process in minimizing latero-deviation, patients were initially divided into two groups based on the presence or lack of the coronoid process. Of the 10 patients, 5 had undergone surgery which had spared the former structure, whilst 5 had lost it during surgery. For the assessment, a comparison was drawn between patients that satisfied two conditions: the presence of teeth and the lack of a reconstruction plate. The presence of dental elements proved essential because they worked as landmarks in quantifying the deviation of the mandible. The presence of the reconstruction plate automatically greatly reduces latero-deviation, and for this reason patients that had undergone insertion of the latter were excluded.

Four patients satisfied the criteria: two with the coronoid process and two without it.

The reader is now invited to focus its attention on the similarities and differences between cases n° 2 and n° 4. Both patients have undergone surgery with closure of wound using the pectoralis major flap and both patients retained lower anterior teeth at the time of surgery. Despite this, the deviation towards the surgical site that patient n° 4 displays is massive compared to that of patient n° 2. This can be explained through the main difference between the two surgeries: in patient n°2, the coronoid process was preserved, but it was sacrificed in patient n° 4. The coronoid process is an insertion site for muscular fascia of both the temporalis muscle and the masseter; the conservation of this site and of its insertions represents a protective factor against the deviation of the mandible, as these muscles contribute to stabilizing the location of the mandible itself. This goes to show how the type of surgery and the amount of hard tissue resected are closely related to the challenges that must be faced in the rehabilitation part of the therapy.

Regarding the extension of the defect, the most important elements are the relationship with the midline (does the defect go over the midline?) and the resection or preservation of pillar structures such as the condyle or coronoid process. In cases where these structures were spared, the deviation of the mandible is much more easily controlled, because of the positive effect due to the remaining masticatory muscles on the affected side that help balance out the latero-deviation. This translates to a decrease in the challenges of dental rehabilitation because the patient can autonomously find and maintain a correct mandible position. As for the midline, none of the patients that were enrolled in this study presented a defect that extended over the midline, but according to literature, resection of the entire anterior arch is best reconstructed with vascularized bone flaps, because this technique allows one to maintain chin projection and nullify the potentially massive deviation that these patients would inevitably present, thus preserving mandibular contour and accurate dental occlusion [7].

The presence of teeth on the surviving mandibular segment can lead to a dental retained mobile prosthesis, which can be considered a compromise between ideal rehabilitation (fixed or removable prosthesis on implant) and barely acceptable rehabilitation (complete removable denture). It can provide sufficient retention to allow efficient chewing on the non-affected side while also providing an extensive flange on the surgical side, which will in turn anchor prosthetic teeth that will determine a good occlusion form.

In completely edentulous patients, the rehabilitation plan can take a different form: carefully planned implants on the surviving segment that may or may not be solidarized through a primary structure (to balance any potential lack of parallelism) that support a removable prosthesis (overdenture). Although the surgery required to insert implants can be arduous due to the general health status of these patients, the major control that may be exercised over the position and inclination of the pillars of the planned prosthesis results in customization of the rehabilitation plane (which is fundamental due to the unique oral characteristics of these patients). Lastly, in patients who are not good candidates for implant surgery, or who simply do not have the means to undergo such an expense, a well-planned and customized complete removable prosthetic may represent an acceptable solution in partially restoring oral functions [31].

## 5. Conclusions

**Figure 25:**
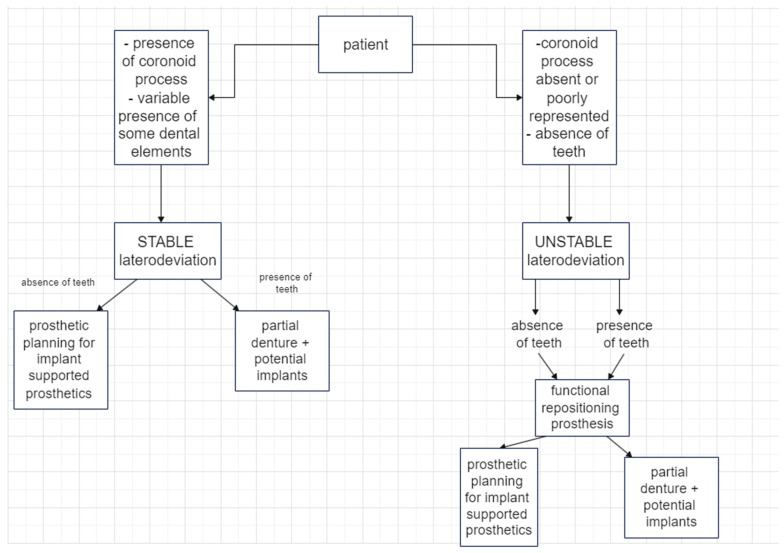
Decision-making algorithm for the rehabilitation of patients with hemimandibulectomy.

The goal of this paper is to show that despite major challenges, the restoration of a correct occlusion form is possible and can be achieved in patients that have undergone hemimandibulectomy without mandibular continuity. This study has also attempted to define the best management to achieve occlusal form, and we propose the following decision-making algorithm, with the goal of giving some guidance to clinicians facing the peculiar issues that patients who undergo hemimandibulectomy present (Figure 25).

In an ideal case scenario, a patient with hemimandibulectomy would be rehabilitated through a bony flap, such as the fibula free flap, which would be modified to better suit the surviving mandibular segments. The bone graft would subsequently be used to support dental implants which would in turn support a prosthesis (generally removable) or a primary structure to solidarize the different implants. This represents a gold standard in dental rehabilitation care; however, this scenario is not always implementable as it would require a young, otherwise healthy patient with no comorbidities. Whenever this is not the case, both physicians and patients must be eager to find a compromise and manage expectations to provide the best possible function and aesthetical result given the situation.

Finally, this paper also highlights the lack of attention surrounding the coronoid structure, its presence or absence and its role in limiting the lateral deviation that occurs naturally. Although further research is needed, it would be promising if the classifications of mandibular defects were to evolve and include the presence or lack of the coronoid as a specific focus.

## Figures and Tables

**Table 1:** Sample characteristics: age/gender, type of surgery and reconstruction modality, adjuvant therapy (radiation and/or chemotherapy or no therapy) and post-surgery dental status. The description of the mandibular defect is based on Brauners’ classification.

N°	Age/Gender	Resection + Reconstruction Modality	Adjuvant Therapy	Denture Status
1	52/M	Hemimandibulectomy (L2-3). Closure of surgical wound with pedunculated pectoralis major (PM) flap	Radiotherapy post op (1 cycle)	41,42,43, 47
2	78/F	Hemimandibulectomy (L1-2-3). Closure of surgical wound with pedunculated PM flap	Radiotherapy post op (1 cycle)	41,42,43,31,32,33,35
3	75/F	Hemimandibulectomy (L2-3). Closure of wound with pedunculated PM flap Fixation plate inserted between condylar segment and symphysis.	Radiotherapy post op	42,43,44,45
4	57/M	Hemimandibulectomy (L2-3). Pelvectomy, closure of wound with pedunculated PM flap	No Therapy	43,44,46
5	76/M	Hemimandibulectomy (L1-2-3). Resection of tonsil pillar and soft tissue of cheek, closure of wound with pedunculated PM flap	No Therapy	Edentulous
6	71/M	Hemimandibulectomy (L1-2-3). Pelvectomy closure of wound with pedunculated PM flap	Chemotherapy and Radiotherapy post op	Edentulous
7	68/F	Hemimandibulectomy (L2). Closure of surgical site through local flaps	No Therapy	Edentulous
8	78/M	Hemimandibulectomy (L-2-3). Fixation plate between the condylar segment and symphysis closure of surgical site with pedunculated PM flap	No Therapy	31,32,33, 41,42,43,44
9	74/M	Hemimandibulectomy (L1-2-3). Surgical wound was closed with pedunculated PM flap.	Radiotherapy post op (1 cycle)	Edentulous
10	71/M	Hemimandibulectomy (L2, L3). Surgical wound was closed with pedunculated PM flap	Radiotherapy post op (1 cycle)	31,32,33,34,41,42

## Data Availability

The original contributions presented in the study are included in the article; further inquiries can be directed to the corresponding author.
